# Developing an Exergame Intervention to Prevent Alzheimer’s Disease

**DOI:** 10.1093/geroni/igaa057.3117

**Published:** 2020-12-16

**Authors:** Fang Yu, Dereck Salisbury, Tom Plocher

**Affiliations:** 1 Arizona State University, Minneapolis, Minnesota, United States; 2 University of Minnesota, Minneapolis, Minnesota, United States; 3 Moai Technologies, Maple Grove, Minnesota, United States

## Abstract

Delaying the onset of Alzheimer’s Disease by five years could save the U.S. ~$89 billion by 2030. Aerobic exercise and cognitive training are two promising interventions for AD prevention and the two together may have a synergistic cognitive effect than either alone. The purposes of this study were to develop an integrated virtual-reality cognitive training (VRCT) and cycling intervention known as exergame and test its feasibility in older adults with subjective cognitive decline (SCD). The VRCT included grocery shopping from a list, flower shopping from a list, dinnerware sorting, book sorting, and postage estimation. Twelve participants enrolled in the 1-month program (12 sessions) achieved 81.2% session adherence and 81.4% adherence to the exercise prescription. The exergame was well accepted by 75% of the participants and 100% were satisfied with the exergame quality and delivery. To conclude, exergame is a flexible intervention that is feasible for older adults with SCD.

